# Early Response to Dexamethasone as Prognostic Factor: Result from Indonesian Childhood WK-ALL Protocol in Yogyakarta

**DOI:** 10.1155/2012/417941

**Published:** 2012-04-03

**Authors:** Pudjo H. Widjajanto, Sutaryo Sutaryo, Ignatius Purwanto, Peter M. vd Ven, Anjo J. P. Veerman

**Affiliations:** ^1^Pediatric Hematology and Oncology Division, Department of Pediatrics, Dr. Sardjito Hospital, Medical Faculty, Universitas Gadjah Mada, Yogyakarta, Indonesia; ^2^Department of Epidemiology and Biostatistics, VU University Medical Center, 1007MB Amsterdam, The Netherlands; ^3^Pediatric Oncology/Hematology Division, Department of Pediatrics, VU University Medical Center, 1007MB Amsterdam, The Netherlands

## Abstract

Early response to treatment has been shown to be an important prognostic factor of childhood acute lymphoblastic leukemia (ALL) patients in Western studies. We studied this factor in the setting of a low-income province in 165 patients treated on Indonesian WK-ALL-2000 protocol between 1999 and 2006. Poor early response, defined as a peripheral lymphoblasts count of ≥1000/*μ*L after 7 days of oral dexamethasone plus one intrathecal methotrexate (MTX), occurred in 19.4% of the patients. Poor responders showed a higher probability of induction failures compared to good responders (53.1% versus 23.3%, *P* < 0.01), higher probability of resistant disease (15.6% versus 4.5%, *P* = 0.02), shorter disease-free survival (*P* = 0.034; 5-year DFS: 24.9% ± 12.1% versus 48.6% ± 5.7%), and shorter event-free survival (*P* = 0.002; 5-year EFS: 9.7% ± 5.3% versus 26.3% ± 3.8%). We observed that the percentage of poor responders in our setting was higher than reported for Western countries with prednisone or prednisolone as the steroids. The study did not demonstrate a significant additive prognostic value of early response over other known risk factors (age and white blood cell count) for DFS and only a moderately added value for EFS.

## 1. Introduction

International studies in the United States and Europe have shown the importance of clinical and biologic characteristics as prognostic factors in childhood ALL. Risk-based treatment has led to a significantly increased cure rate to more than 80% [1], and poor early response to treatment has been well known as a strong predictor for adverse outcome in childhood ALL [2–9]. Poor responder patients with persistence of lymphoblasts of 1,000/*μ*L or more in the peripheral blood on day 8 or 25% or more in marrow on day 14 of treatment show poorer outcomes than the good responder patients [2, 10, 11]. This measurement allows early detection of a patient subpopulation at high risk that is not classified as such by National Cancer Institute (NCI), Rome criteria for risk stratification [12]. Advance technique that refines morphology examination in assessing treatment response is minimal residual disease (MRD) measurement. MRD-based stratification was proven predictive for clinical outcomes in some large series study [13–17].

In comparison with marrow aspiration, peripheral blast measurement is much more practical, less invasive, and cheaper, while MRD evaluation using flow cytometric or PCR method is not available in low-income countries. There is a lack of data related to the measurement of the early response to treatment as predictor for adverse outcome obtained from developing countries, all come from Western countries. We therefore conducted this study in Yogyakarta, Indonesia in the setting of a low-income country with cure rates in the order of 20–30% in patients treated with a dexamethasone-based protocol.

## 2. Materials and Methods

### 2.1. Patients

This study enrolled newly diagnosed childhood ALL patients treated with WK-ALL-2000 protocol in the Pediatric Cancer Unit of the Dr. Sardjito Teaching Hospital (DSH) of the Universitas Gadjah Mada, Yogyakarta, Indonesia during the period of February 1999 to March 2006. The diagnosis of ALL was based on morphology and cytochemistry of marrow specimens that were assessed by two experienced technicians. The inclusion criteria were age range 0–14, FAB morphology L1 or L2, no previous treatment with steroids or leukemia treatment and parents approval. Patients were classified into the high-risk (HR) group based on age of less than 1 year or more than 10 years, WBC count 50,000/*μ*L or more, and presence of mediastinal mass or central nervous system (CNS) involvement. The others were classified as standard risk (SR) group. Treatment started with one-week dexamethasone (6 mg/m^2^) plus one intrathecal MTX on day 1 (dose according to age). Patients were moved from SR to HR group and treated accordingly when they showed poor early response to treatment. Of 209 newly diagnosed childhood ALL patients admitted to DSH, 30 patients were excluded because they did not meet inclusion criteria (21) or moved to another protocol during treatment (9). Of the remaining 179 eligible patients, 14 patients were excluded because they did not have data regarding early response to treatment due to early death caused by toxic condition of leukemia (9) or abandonment (3) before evaluation of early response was performed and the measurement was not conducted in 2 patients. Analysis was done on 165 patients at the end of observation on July 31, 2010. 

### 2.2. Treatment Protocol

**Figure 1:**
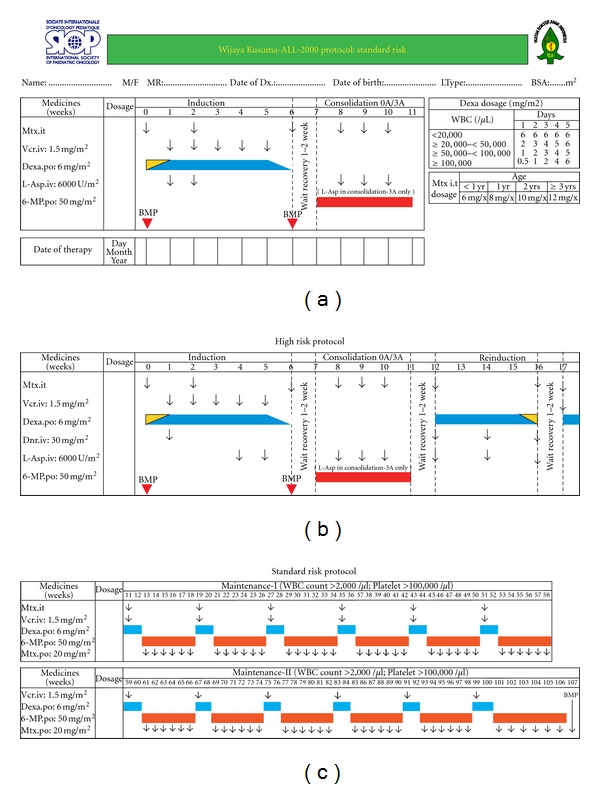
The Indonesian WK-ALL-2000 protocol for childhood ALL. Induction and consolidation treatment of standard risk protocol (a). One dose of daunorubicin (Dnr) in induction plus a reinduction treatment was added in high-risk protocol (b). The maintenance treatment was similar between the standard risk and high risk protocols (c). 3A/0A were group of patients who received/did not receive 3 extra doses of L-asparaginase during consolidation treatment.

All patients were treated on WK-ALL-2000 protocol (Figure 1). It was the first Indonesian national protocol for childhood ALL [18] adapted from the dexamethasone-based Dutch protocol ALL-VI [19]. The protocol consisted of 1-week prephase, continued with 5-week induction, 4-week consolidation and blocks in maintenance treatment of 95 weeks in SR patients. HR patients received additional one dose of daunorubicin in induction treatment plus a reinduction treatment (4 weeks) that was inserted after the consolidation treatment, otherwise the drugs and schedule were similar between the HR and SR protocols. Infants less than 1 year of age were grouped and treated as HR because a separate protocol for this group of patients was not yet developed in our institution. Patients were also randomized to receive or not to receive 3 extra doses of L-Asparaginase during consolidation treatment (manuscript in preparation). 

### 2.3. Early Response to Treatment Measurement

Early response to treatment was measured as an absolute number of peripheral lymphoblasts at day 8 of induction treatment, after administration of 7 days oral dexamethasone 6 mg/m^2^, but adjusted to WBC count to prevent tumor lysis syndrome. Patients with a WBC count at diagnosis between 20,000/*μ*L–50,000/*μ*L started with 2 mg/m^2^ dexamethasone, and increased the dose in 4 days to 6 mg/m^2^; WBC count between 50,000/*μ*L–100,000/*μ*L started with 1 mg/m^2^ dexamethasone and increased the dose in 5 days to 6 mg/m^2^. Patients with WBC count of more than 100,000/*μ*L started at 0.5 mg/m^2^. One dose of age-adjusted intrathecal MTX was scheduled on day 1, that was 6, 8, 10 or 12 mg for age less than 1, ≥1-2, ≥2-3 or ≥3 year(s), respectively. In our situation the intrathecal MTX administration was not given on day 1 of treatment in some cases due to unsuccessful lumbar puncture or drug availability. Analysis of patients record learned that intrathecal MTX was given on days 0–2 in 43%, days 3–5 in 20%, after day 5 in 9% of patients, and in 28% the day of administration was not noted in the patients record. Patients were classified as good responders when the absolute peripheral lymphoblasts count at day 8 was less than 1,000/*μ*L and poor responder when 1,000/*μ*L or more. 

### 2.4. Outcome Variables and Statistical Analysis

 The outcome variables evaluated were complete remission (CR) achievement, death, abandonment of treatment, and development of relapse. Complete remission was determined at the end of induction (day 42 of treatment) as the absence of lymphoblasts in peripheral blood and cerebrospinal fluid, less than 5% lymphoblasts in active hematopoetic marrow with no evidence of localized disease anywhere. Resistant disease was defined as failure to achieve CR. Abandonment of treatment was ascertained when treatment was initiated but not completed. Relapse was defined as recurrence of lymphoblasts or localized infiltration of lymphoblasts at any site after CR. Disease-free survival (DFS) was calculated as the interval between date of entering treatment and date of failure to achieve CR (resistant disease) or to a relapse. Patients who abandoned treatment were considered failures at date of abandonment and were censored at the time they were last seen. Event-free survival (EFS) was calculated as the interval between date of entering treatment and date of an event that occurred first: induction failure, death, abandonment, or relapse. Induction failures consist of death and abandonment during induction and resistant disease. DFS and EFS analyses were performed using Kaplan-Meier method and survival of poor responders and good responders were compared using the log rank test. Possible risk factors were first identified in univariate survival analyses. All risk factors with a *P* value less than 0.10 in the univariate analyses were included in a multivariate analysis. The multivariate analysis was done using Cox regression. All data were analyzed using Statistical Package for Social Sciences (SPSS) program version 13, and a two-sided *P* value less than.05 was used as level for statistically significance.

## 3. Results

 Characteristics of the patients are shown in Table 1. Poor responder patients were 32 (19.4%) of 165 patients, consisting of 7 SR patients who moved to HR group on day 8 of treatment, 3 standard risk patients who remained in SR group by mistake, and 22 patients who fulfilled already HR criteria at diagnosis. A higher percentage of poor responders as compared to good responders fell into HR group (68.8% versus 32.3%, *P* < 0.01), age category 10–14 years (34.4% versus 11.3%, *P* < 0.01), WBC count at diagnosis category ≥ 50,000/*μ*L (56.2% versus 16.5%, *P* < 0.01), and percentage of peripheral blasts count at diagnosis category 50–100% (78.1% versus 50.4%, *P* < 0.01). The median WBC count at diagnosis was much higher in poor responder patients (70,100 (range: 2,000–424,000) versus 7,800 (range: 600–346.000) per *μ*L, Mann-Whitney *U* test, *P* = 0.01). Treatment outcome by groups of early response to treatment measured at day 8 is shown in Table 2. Complete remission was achieved in 117 (70.9%) of 165 patients: 15 (46.9%) of 32 in poor responder and 102 (70.9%) of 133 in good responders patients. Poor responders were more likely to experience induction failures than good responders (OR = 3.73, 95% CI 1.67–8.32, *P* = 0.001). A post hoc analysis for comparing the probability of occurrence of the different type of induction failures between poor and good responder groups showed a higher odds for resistant disease and death (both relative to complete remission) in the poor responder group (*P* = 0.01 and *P* = 0.03, resp.). The odds for abandonment did not differ between the groups (*P* = 0.09). 

After achieving CR, the most relevant adverse event was relapse (38.5%), followed by death in remission (16.2%) and abandonment (14.5%). Although not statistically significant, relapse and death in remission were higher in poor responders than in good responder patients. Twenty-nine (65%) of all 45 relapse cases occurred during treatment, mostly in the maintenance phase in 25 (55%) of cases. Thirty-three (73%) relapses occurred as isolated hematological relapses. 

**Figure 2:**
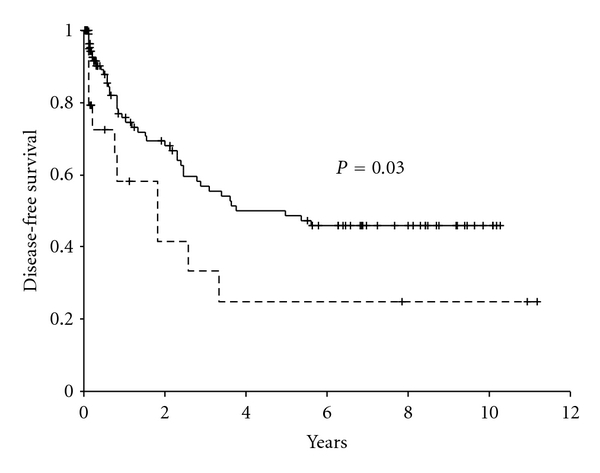
Kaplan-Meier survival curve for disease-free survival by early response to dexamethasone treatment. The events are resistant disease and relapses. The continuous line represents good responders (*n* = 133) and the dashed line represents poor responders (*n* = 32).

**Figure 3:**
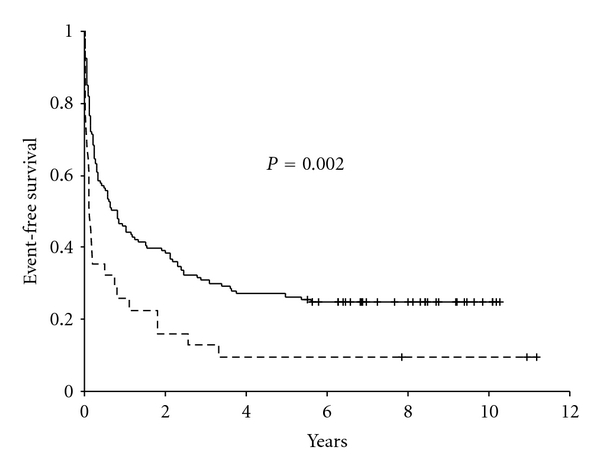
Kaplan-Meier survival curve for event-free survival by early response to dexamethasone treatment. The events are abandonment or death during induction and after complete remission, also resistant disease and relapse. The continuous line represents good responders (*n* = 133) and the dashed line represents poor responders (*n* = 32).

When establishing the relation between early response to treatment and survival using Kaplan-Meier analyses it was found that poor responder patients had a significantly lower DFS (*P* = 0.034) and EFS (*P* = 0.002) (Figures 2 and 3). The 5-year DFS probability was 24.9% ± 12.1% for poor responders compared to 48.6% ± 5.7% for good responders. The 5-year EFS probabilities were 9.7% ± 5.3% and 26.3% ± 3.8% for poor responders and good responders, respectively. 

Univariate analysis showed that age at diagnosis, WBC count at diagnosis and early response to treatment were predictive for DFS and EFS (Tables 3 and 4). Further multivariable analysis using forward selection (with age at diagnosis, WBC count, and early response) revealed that age at diagnosis remained as the only independent prognostic factor for DFS (HR 2.63, 95% CI 1.32–5.26, *P* = 0.006) as presented in Table 3. 

Age at diagnosis and WBC count were identified as independent prognostic factors for EFS (HR for age 1.75, 95% CI: 1.10–2.79, *P* = 0.018; HR for WBC count 1.77, 95% CI: 1.18–2.66, *P* = 0.006), see Table 4. When early response was added to these models the association with survival was not significant for DFS and only borderline significant for EFS (DFS: HR for early response corrected for age 1.75, 95% CI: 0.89–3.46, *P* = 0.105; EFS: HR for early response corrected for age and WBC count category 1.53, 95% CI: 0.98–2.39, *P* = 0.062), indicating that part of the association between early response and survival that was found in the univariate analyses could be explained through its association with other risk factors. 

## 4. Discussion

 Early response to treatment has been associated with prognosis in childhood ALL [2–5, 9, 20, 21] as well as in subpopulations of ALL such as infants [22], T-cell ALL [23, 24], and Philadelphia chromosome-positive (Ph+) ALL [25, 26]. All studies revealed that poor responders with persistence of a relatively high number of lymphoblasts in the peripheral blood after 7 days treatment or in bone marrow after 14 days treatment had significantly poorer outcome than good responder patients. A study by Rautonen et al. [27] revealed that the longer the time required for disappearance of peripheral lymphoblasts, the greater the risk for adverse events. 

A Dutch study showed that laboratory* in vitro* resistance to prednisolone was associated with a worse outcome [28]. The frequency of poor responder patients with peripheral lymphoblasts 1000/*μ*L or more after 7-day prednisone treatment and 1 dose of intrathecal MTX at day 1 ranged from 7.5% to 15% in European studies [29, 30], while in our study the poor responder rate was 19%. The difference may be explained by delay in administrating intrathecal MTX, different steroid, slower steroid escalation, and high percentage of early death and abandonment in our study. The steroid used in our study was dexamethasone 6 mg/m^2^ instead of prednisone 60 mg/m^2^ or prednisolone as used in those the Western country. In our situation the intrathecal MTX administration was not always given on day 1 as scheduled. A European Organization for Research and Treatment of Cancer (EORTC) study published by Thyss et al. [31] showed that postponing intrathecal MTX injection generated an increasing percentage of poor responder patients, that was 9.6% if intrathecal MTX injection was given before day 2, but rose to 23.1% if it was given between day 2 and day 6, and even to 30% if it was given at day 6 or after. So, we assumed that the noticeable higher number of poor responder patients in our study may have been due to postponing intrathecal MTX injection. The role of the lymphoblast lineage is also important in the response to steroids and MTX treatment [23, 24]. Laboratory *in vitro *studies reveal that T-cell ALL has lower sentivity to corticosteroids and MTX than B-cell ALL [32, 33]. Whether the poor responders in our study were more often T-ALL cases could not be confirmed due to lack of immunophenotypic data, which were not yet available during the Indonesian WK-ALL-2000 protocol. 

Studies in Western countries revealed that the poor responder patients were significantly associated with unfavorable outcome with respect to DFS and EFS [11]. Our study confirmed it for EFS only and not for DFS. Univariate analysis shows that poor response is associated with increased chance of induction failures and worse DFS and EFS, suggesting that the measurement could be used in a risk-based treatment for childhood acute lymphoblastic leukemia. However, this study failed to demonstrate a significantly added prognostic value for this factor over other known risk factors for DFS and only a moderate added prognostic value for EFS. One has to bear in mind however, that we used poor response as a stratification factor, that is, the poor responders got a more intensive treatment, which might have resulted in higher DFS. For DFS age remained the most important risk factor, whereas age and WBC count were identified as significant predictors for EFS. So we suggest that all these factors together with early response are taken into account in a risk-based treatment for childhood acute lymphoblastic leukemia. Our study confirmed the role of morphology in evaluating the early response to treatment for countries with limited economic resources and with high rate of treatment abandonment contributed significantly for treatment outcomes. 

Significantly higher induction failures (including death and abandonment) and resistant disease at the end of induction were shown in the poor responder group. Although the poor responders did worse in induction, our study failed to show significantly that poor responder patients had a higher relapse rate and shorter period to experience relapse as first event after achieving remission. In comparison to the Dutch study with relapses in 14% cases [34], our study showed an almost 3-fold higher relapse rate. This could be explained due to our less intensive protocol but particularly due to low adherence to the protocol [35, 36]. Quite often there was no medicine available or no money for medicine, so many patients got (far) less than the scheduled doses especially in the maintenance phase. Our less intensive protocol and low adherence to it may explain why most relapses occurred during treatment in our study. Low EFS in our study was also caused by a high toxic death rate (22%, 36/165) and abandonment (22%, 37/165) during induction and remission. Both adverse events have a significant contribution to failure in our treatment protocol as reported by Mostert et al. [37] and studies from other low-income countries [38, 39]. Social and economical factors related to poor protocol compliance were also reported in our hospital [35–37]. This is contrary to the western countries where toxic death is approximately 2–5% and abandonment is virtually unknown [40, 41]. 

We conclude that the frequency of poor responder patients in Indonesia with dexamethasone as steroid and one dose of intrathecal MTX is higher than found in most large and well-reported Western studies. It has a strong predictive value for induction failure and for resistant disease and is associated with poorer outcome than the good responder patients. This measurement is practical, noninvasive, and affordable. Early response to treatment is thus not only in high income countries, but even more in low-income country a very useful means of improving stratification for children with ALL in addition to the NCI-Rome criteria.

## Figures and Tables

**Table 1:** Characteristics of patients.

	Poor responder (*N* = 32)		Good responder (*N* = 133)		Total (*N* = 165)		*P* value
	*n*	(%)	*n*	(%)	*n*	(%)	
Risk group							
Standard risk	10^a^	*31.3*	90	*67.7*	100	*60.6*	<0.01
High risk	22	*68.8*	43	*32.3*	65	*39.4*	

Gender							
Girl	14	*43.8*	54	*40.6*	68	*41.2*	0.75
Boy	18	*56.3*	79	*59.4*	97	*58.8*	

Age group (NCI criteria)^b^							
Standard risk	21	*65.6*	118	*88.7*	139	*84.2*	<0.01
High risk	11	*34.4*	15	*11.3*	26	15.8	

WBC count (/*μ*L)							
<50,000	14	*43.8*	111	*83.5*	125	*75.8*	<0.01
≥50,000	18	*56.2*	22	*16.5*	40	*24.2*	
Median (range)	70,000 (2,000–424,000)		7,800 (600–346,000)				

Peripheral lymphoblasts count at diagnosis (%)^c^							
0–49	7	*21.9*	65	*49.6*	72	*44.2*	<0.01
50–100	25	*78.1*	66	*50.4*	91	*55.8*	

^a^Of 10 standard risk patients with poor response, 7 moved to high risk and 3 were still in standard risk by mistake. ^b^Standard risk (1–9 years), high risk (10–14 years and infant <1 year (including 1 patient)). ^c^Data not available in 2 patients.

**Table 2:** Treatment outcome by groups of early response to treatment measured at day 8.

	Good responder (*N* = 133)		Poor responder (*n* = 32)		Total (*N* = 165)		OR	95% CI	*P* value
	*n*	(%)	*n*	(%)	*n*	(%)			
Induction treatment									
Complete remission	102	*76.7*	15	*46.9*	117	*70.9*			
Induction failures	31	*23.3*	17	*53.1*	48	*29.1*	3.73^a^	1.67–8.32	0.001^1^
Abandonment	14	*10.5*	6	*18.8*	20	*12.1*	2.91^a^	0.97–8.75	0.09^2^
Death	11	*8.3*	6	*18.8*	17	*10.3*	3.71^a^	1.19–11.51	0.03^2^
Resistant disease	6	*4.5*	5	*15.6*	11	*6.7*	5.67^a^	1.54–20.89	0.01^2^

Adverse event after CR									
Continuous CR	33	*32.4*	3	*20.0*	36	*30.8*			
Relapse	38	*37.3*	7	*46.7*	45	*38.5*	2.02^b^	0.48–8.47	0.50^2^
Death	16	*15.7*	3	*20.0*	19	*16.2*	2.06^b^	0.37–11.38	0.40^2^
Abandonment	15	*14.7*	2	*13.3*	17	*14.5*	1.47^b^	0.22–9.71	0.65^2^

Site of relapse	38		7						
Isolated hematological	28	*73.7*	5	*71.4*	33	*73.4*			
Isolated CNS	7	*18.5*	1	*14.3*	8	*17.8*			
Other	3	*7.8*	1	*14.3*	4	*9.8*			

OR: odds ratio for poor responders relative to good responders; CI: confidence interval; CR: complete remission. ^a^ORs for any induction failure and specific induction failures (CR during induction is taken as the reference outcome category). ^b^ORs for first event after CR (continuous CR is taken as the reference outcome category). ^1^Chi-square test. ^2^Fisher exact test.

**Table 3:** Univariate and multivariate analyses of factors predicting disease-free survival.

Factors	HR	95% CI	*P* value
*Univariate analyses*			
Age at diagnosis	2.64^1^	1.32–5.26	0.006
WBC group at diagnosis	1.99^2^	1.06–3.74	0.03
Early response to treatment	2.02^3^	1.04–3.92	0.04
Gender	0.89^4^	0.52–1.53	0.67
*Multivariate analysis* ^ a^			
Age at diagnosis	2.63	1.32–5.26	0.006

^a^Final model. Age at diagnosis, WBC group, and early response were included as factors in a stepwise analysis. Note that all three were also factors to stratify patient into the HR group. The same model was found using forward selection.

^1^For age <1 and ≥10 years relative to 1–9 years.

^2^For WBC ≥ 50,000 relative to WBC < 50,000.

^3^For poor responders relative to good responders.

^4^For girls relative to boys.

**Table 4:** Univariate and multivariate analyses of factors predicting event-free survival.

Factors	HR	95% CI	*P* value
*Univariate analyses*			
Age at diagnosis	2.05^1^	1.31–3.22	0.02
WBC group at diagnosis	1.98^2^	1.34–2.92	0.01
Early response to treatment	1.91^3^	1.25–2.91	0.03
Gender	1.03^4^	0.72–1.46	0.87
*Multivariate analysis* ^ a^			
Age at diagnosis	1.75	1.10–2.79	0.018
WBC group at diagnosis	1.77	1.18–2.66	0.006

^a^Final model. Age at diagnosis, WBC group, and early response were included as factors in a stepwise analysis. All three were also used for stratification into HR group. The model was found using forward selection.

^1^For age <1 and ≥10 years relative to 1–9 years.

^2^For WBC ≥ 50,000 relative to WBC < 50,000.

^3^For poor responders relative to good responders.

^4^For girls relative to boys.
